# Control of cell migration in the development of the posterior lateral line: antagonistic interactions between the chemokine receptors CXCR4 and CXCR7/RDC1

**DOI:** 10.1186/1471-213X-7-23

**Published:** 2007-03-29

**Authors:** Christine Dambly-Chaudière, Nicolas Cubedo, Alain Ghysen

**Affiliations:** 1 Lab of Neurogenetics INSERM U881, Montpellier, France; Université Montpellier II, Montpellier, France

## Abstract

**Background:**

The formation of the posterior lateral line of teleosts depends on the migration of a primordium that originates near the otic vesicle and moves to the tip of the tail. Groups of cells at the trailing edge of the primordium slow down at regular intervals and eventually settle to differentiate as sense organs. The migration of the primordium is driven by the chemokine SDF1 and by its receptor CXCR4, encoded respectively by the genes *sdf1a *and *cxcr4b*. *cxcr4b *is expressed in the migrating cells and is down-regulated in the trailing cells of the primordium. *sdf1a *is expressed along the path of migration. There is no evidence for a gradient of *sdf1a *expression, however, and the origin of the directionality of migration is not known.

**Results:**

Here we document the expression of a second chemokine receptor gene, *cxcr7*, in the migrating primordium. We show that *cxcr7 *is highly expressed in the trailing cells of the primordium but not at all in the leading cells, a pattern that is complementary to that of *cxcr4b*. Even though *cxcr7 *is not expressed in the cells that lead primordium migration, its inactivation results in impaired migration. The phenotypes of *cxcr4b*, *cxcr7 *double morphant embryos suggest, however, that CXCR7 does not contribute to the migratory capabilities of primordium cells. We also show that, in the absence of *cxcr4b*, expression of *cxcr7 *becomes ubiquitous in the stalled primordium.

**Conclusion:**

Our observations suggest that CXCR7 is required to provide directionality to the migration. We propose that directionality is imposed on the primordium as soon as it comes in contact with the stripe of SDF1, and is maintained throughout migration by a negative interaction between the two receptors.

## Background

Directed cell migration is involved in many aspects of development including the establishment of the embryonic body plan, organogenesis and organ function. It also plays a role in several pathological processes, notably the spread of tumour cells and formation of metastases. Identification of the molecules governing cell migration is therefore of major importance. Most work on cell migration relies on *in vitro *systems where migration is relatively easy to monitor and quantify. This has led to substantial progress in understanding the cell biology of migration as well as the many receptor molecules and signaling cascades involved. Migration is crucially dependent on the cell environment, however, and ideally one would like to study its control in a system where migration can be visualized *in vivo *and in real time.

The lateral-line system of the zebrafish has emerged recently as a useful model for studying the process of long-distance cell migration and for unraveling its genetic control [1]. The lateral-line is a mechanosensory system used by fish to detect water movements and plays an important role in a variety of behaviours [2]. It comprises discrete sense organs, the neuromasts, arranged on the body surface in species-specific patterns. The posterior lateral line (PLL), which extends on the trunk and tail, comprises at the end of embryogenesis a line of five neuromasts regularly spaced along the trunk and tail, and a cluster of two-three terminal neuromasts at the tip of the tail [3]. This pattern is widely conserved among teleost embryos [4].

All neuromasts of the PLL originate from a sensory placode that forms just posterior to the otic vesicle [5,6]. A group of about 100 cells delaminate from the placode to form a migrating primordium that moves all the way to the tip of the tail at a constant speed of 1.7 somite/h [7]. The journey lasts 20 h, from 20 to 40 hpf, and the migrating primordium deposits in its wake five groups of cells that will become the neuromasts L1 – L5. Migrating cells keep their relative positions within the migrating primordium, and each deposition results from a progressive slowing down of a group of around 20 cells at the trailing edge [7,8]. Once these 20 cells have settled down, they differentiate as hair cells and support cells to form a neuromast. Neuromasts are connected by a thin stripe of interneuromastic cells that also arise from the migrating primordium; these cells will later form intercalary neuromasts [9,10]. Upon reaching the tip of the tail the primordium fragments in 2–3 groups that will form the terminal neuromasts [7].

The primordium is guided along a trail of cells that express the chemokine SDF1, and its migration depends on the partner of SDF1, the chemokine receptor CXCR4 [11,12]. One of the two genes coding for this receptor, *cxcr4b*, is expressed in the migrating cells and is down-regulated in the cells at the trailing edge of the primordium [7]. The inactivation of *sdf1a *in morphant embryos, or of *cxcr4b *in mutant or morphant embryos, results in an arrest of migration [11,12]. A similar effect of *cxcr4b *inactivation has been observed in a mutant line of the more derived fish *Oryzias latipes *(medaka) [13]. Medaka belongs to the neoteleost lineage, while the zebrafish belongs to the more primitive ostariophysian lineage. This suggests that not only the early pattern of the PLL but also the underlying mechanism is highly conserved among teleosts.

In an attempt to discover other elements that contribute to the control of migration we have examined other genes that display heterogeneous patterns of expression within the migrating primordium. Here we report the description of another chemokine receptor, CXCR7. Although long considered an orphan receptor, CXCR7 has recently been shown to recognize SDF1 [14] and possibly other ligands as well [15]. We show that CXCR7 plays an essential role for primordium migration in spite of not being expressed in the vast majority of the migrating cells, and we propose that it is required to provide migration directionality.

## Results

### Identification of *cxcr7*, a gene potentially involved in the control of PLL primordium migration

**Figure 1:**
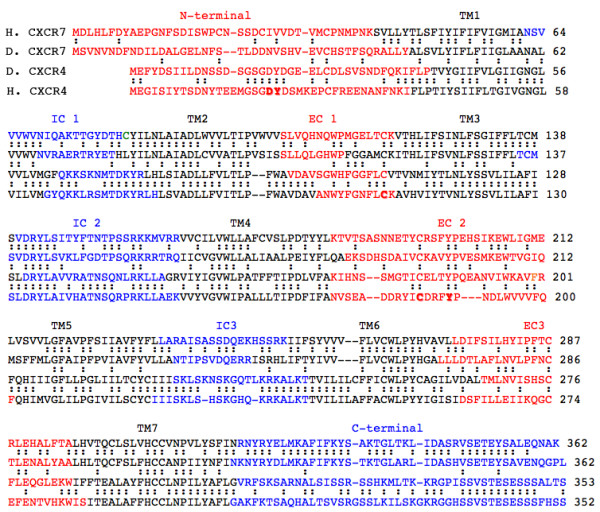
Comparison of predicted sequences of human (*Homo*, H) and fish (*Danio*, D) CXCR4 and CXCR7. Predicted transmembrane domains (TM1-7) are in black, extracellular domains (aminoterminal and EC 1–3) are in red and intracellular domains (IC 1–3 and carboxyterminal) are in blue. Amino-acid identities are indicated between the two fish receptors, as well as between each fish receptor and its human counterpart. Bold letters are residues that are discussed in the "Results".

We recognized *cxcr7 *as an EST potentially involved in the control of PLL primordium migration based on its pattern of expression ([16] and see Material and Methods). Sequence comparisons revealed that this EST corresponds to a gene that encodes the homolog of the mammalian chemokine receptor, CXCR7 (also called RDC1). The putative product of the *Danio *gene is 54% identical to the human CXCR7. Its predicted seven transmembrane domains match reasonably well with those of the human receptor as well as with those of the fish and human CXCR4 receptors (Fig. 1). Some key features of this family of receptors are fully conserved, specifically the putative C109 – C196 disulfide bridge and the nearby tyrosine Y190 which is thought to play an essential role in the conformational change of the receptor upon ligand binding [17].

SDF1 has been shown to bind to the N-terminal, extracytoplasmic domain of CXCR4 [18]. A small stretch of 6 aminoacids is conserved between human and fish CXCR4, of which 2 (D20 Y21) have been shown in *Homo *to be important for the binding of HIV. Besides this short motif, however, there is very little sequence conservation between the N-terminal domains of human and fish CXCR4. There is even less N-terminal conservation between fish CXCR4 and CXCR7, or between fish and human CXCR7 (Fig. 1). The remarkable lack of conservation of the SDF1-binding domain suggests that the recognition of SDF1 is not based on conventional stereochemical matching. This conclusion is fully consistent with the observation that a D-amino-acid version of SDF1 binds to the human CXCR4 receptor as well or even better than the normal L-version [19].

Contrary to the poor conservation of the N-terminal extracellular region, the predicted C-terminal intracellular domain of human and fish CXCR7 are 73% identical. The level of identity is somewhat lower between the human and fish CXCR4 (55%). Interestingly, however, there is essentially no conservation between the C-terminals of the two receptors (an amazingly low 6% in either fish or human), strongly suggesting that CXCR4 and CXCR7 act through different cytoplasmic effectors and play different roles in the control of migration.

### *cxcr7 *expression in the PLL primordium

**Figure 2:**
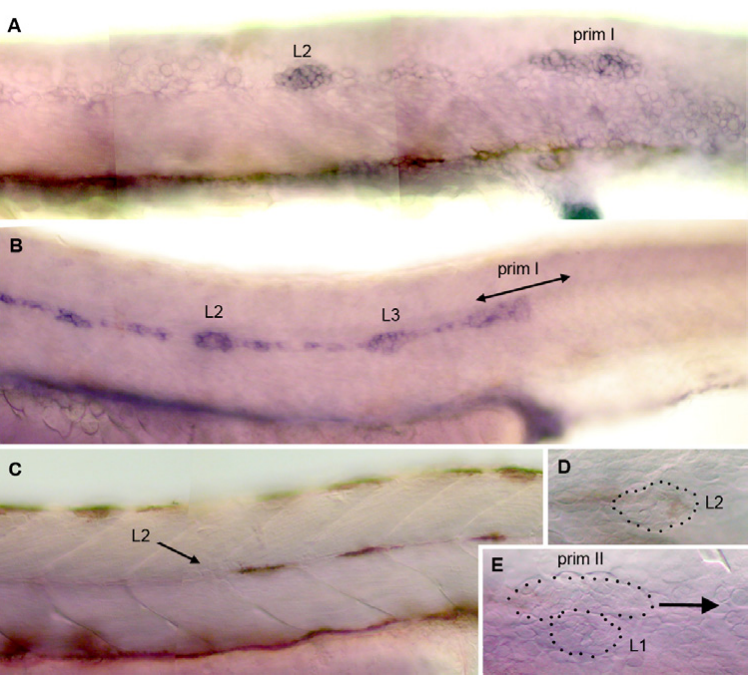
Patterns of *cxcr7 *expression in the developing PLL. A: expression of *claudin *[34] reveals the migrating primordium primI and a freshly deposited proneuromast, L2, in a 32 hpf embryo. B: *cxcr7 *is expressed in the trailing cells of the migrating primordium but not in the leading cells. It is also expressed in proneuromasts L2 and L3 in a 32 hpf embryo, as well as in the trail of interneuromastic cells. C-E: *cxcr7 *expression has disappeared at 48 hpf, both in the interneuromastic cells and in primary neuromasts. E: *cxcr7 *is not detectably expressed in the secondary primordium, primII, which is in the process of migrating past L1 at 48 hpf. In all panels the primordium migrates to the right. The dots in D, E outline deposited neuromasts and migrating primordium.

The PLL placode is first detected around 19 hpf (hours post fertilization) and segregates in a static cell mass that becomes the sensory ganglion, and a migrating primordium that moves posteriorwards [20]. The primordium reaches the level of the anus at about 30 hpf (Fig. 2A) and the tip of the tail at 40 hpf.

The expression of *cxcr7 *during primordium migration was assessed by *in situ *hybridization in whole mount embryos. Expression of *cxcr7 *is confined to the trailing cells of the migrating primordium (Fig. 2B, 3A). Expression is maintained in the cells during and after deposition, both in the clusters of cells that will form the neuromasts and in the trail of interneuromastic cells. The expression of *cxcr7 *in deposited cells is transient, however, and has completely disappeared at 48 hpf (Fig. 2C, 2D).

**Figure 3:**
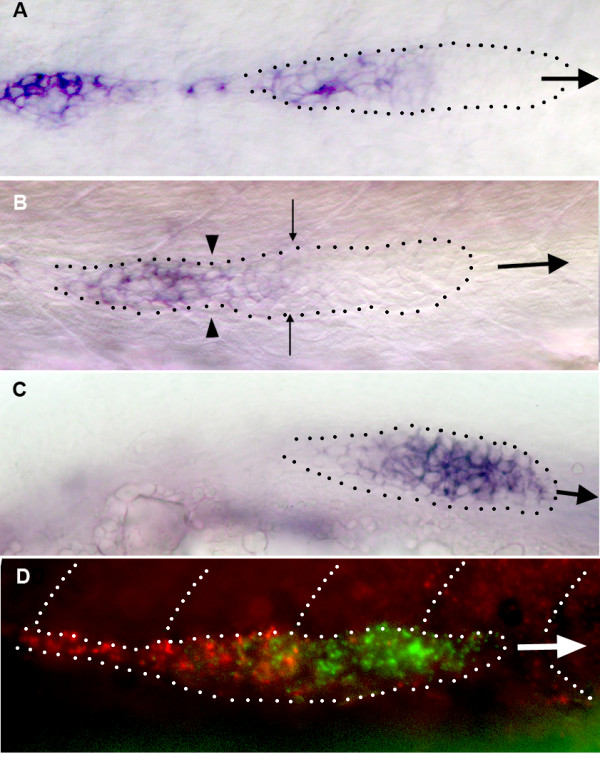
Patterns of gene expression in migrating primordia. A: the expression of *cxcr7 *is strong in the trailing third of primI and absent in the leading half. B: neuromast deposition depletes the cells that express *cxcr7 *most strongly (left of the arrowheads) but cells at the trailing edge of the primordium also express *cxcr7*, albeit at a lower level. The expression of *cxcr7 *must be quickly up-regulated in these cells to re-establish the pre-deposition pattern (compare B and A). C: the expression of *cxcr4 *is high in most of the primordium but weaker in the trailing cells. D: a double *in situ *hybridization reveals that the patterns of expression of *cxcr7 *(red) and *cxcr4b *(green) are largely complementary but not exclusive. In all panels the large arrow shows the direction of primordium migration and the dots outline the migrating primordium. In panel B the arrowheads indicate the limit between the group of cells that are slowing down to form a neuromast, and those that will keep migrating. The fine arrows indicate the boundary of the region of *cxcr7 *expression.

Shortly after primI has reached the tip of the tail and formed the terminal neuromasts, a second primordium arises. This primordium, primII, migrates along the same path as primI and deposits a second wave of about 5 neuromasts [21,22]. The migration of primII is slower than that of primI, and the neuromasts of the second wave are more closely packed. Their polarity is orthogonal to that of the primary neuromasts deposited by primI [23]. We examined the expression pattern of *cxcr7 *at 2 days, when primII has reached somite 7 on average. We observed no *cxcr7 *expression in primII (Fig. 2E).

Besides the PLL, *cxcr7 *is expressed in other discrete regions, notably in parts of the hindbrain, midbrain, forebrain (diencephalon), nose, eye, and kidneys (not shown). In most places the pattern of expression of *cxcr7 *appears highly dynamical.

### Expression of *cxcr7 *and *cxcr4 *during primordium migration

The gene *cxcr7 *is expressed in the trailing part of the primordium, that is, in the cells that are about to be deposited (Fig. 3A). It might be, therefore, that *cxcr7 *expression is lost in the migrating primordium just after deposition. We examined the transitional pattern when the cells with a strong expression of *cxcr7 *are slowing down. We observed in all cases that the cells at the new trailing edge weakly express *cxcr7 *(Fig. 3B) and that this weak expression quickly increases after deposition (Fig. 3A). Thus the expression of *cxcr7 *in trailing cells is not re-initiated after each deposition, but amplified to maintain a dynamical asymmetry within the primordium.

The pattern of expression of *cxcr7 *in the migrating primordium is almost complementary to the pattern reported for *cxcr4b *[7]. The gene *cxcr4b *codes for the chemokine receptor CXCR4 which plays an essential role in the migration of the PLL primordium [11,12]. *cxcr4b *is strongly expressed in the leading two thirds of the primordium and its expression is down-regulated in the trailing third (Fig. 3C). In order to better define the relation between the two patterns we did a double in situ hybridization experiment (Fig. 3D). We observed that the domains of expression of *cxcr7 *and of *cxcr4b *are largely but not completely exclusive, as there is some overlap of expression in the trailing cells. Thus a high level of expression of *cxcr4b *seems to exclude the expression of *cxcr7*, but a high level of *cxcr7 *expression does not preclude the expression of *cxcr4b*.

### Early expression of *cxcr7*, *cxcr4b*, and *sdf1a*

**Figure 4:**
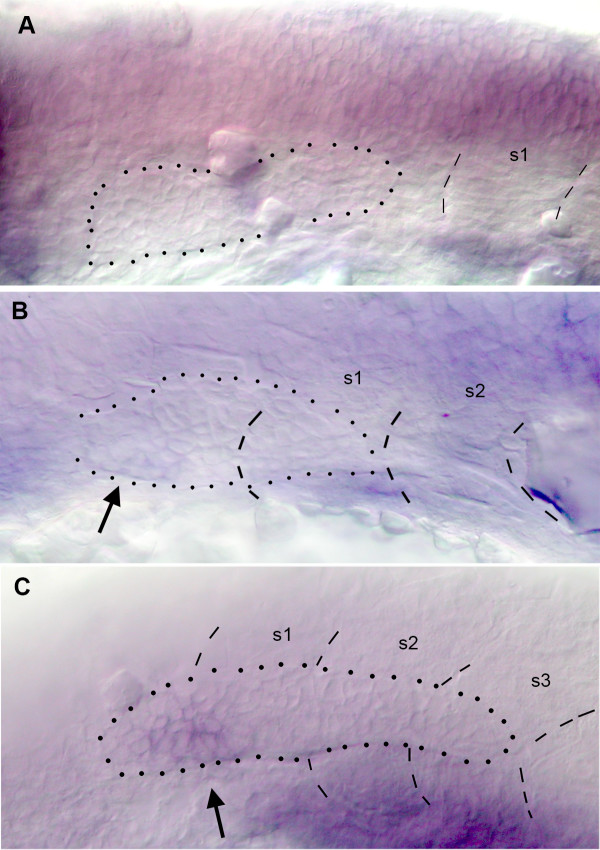
Early expression of *cxcr7*. A: no expression can be detected in the placode or in the primordium that begins to elongate towards the first somite at 20 hpf. B: expression can first be detected in trailing cells (arrow) when the primordium begins to extend along the myoseptum. C: expression increases (arrow) as the primordium extends along somites 1–3.

In order to understand how the patterns of expression of *cxcr7 *and *cxcr4b *are established we performed in situ hybridization on 19–22 hpf embryos. No expression of *cxcr7 *can be detected in the delaminated primordium prior to the onset of migration (Fig. 4A). The earliest expression of *cxcr7 *was detected at about 22hpf, when the leading cells of the primordium are beginning to extend along the horizontal myoseptum. At this time the expression is already confined to the trailing region of the PLL primordium (Fig. 4B, arrow). The expression progressively increases as the primordium migrates over the somites (Fig. 4C, arrow).

**Figure 5:**
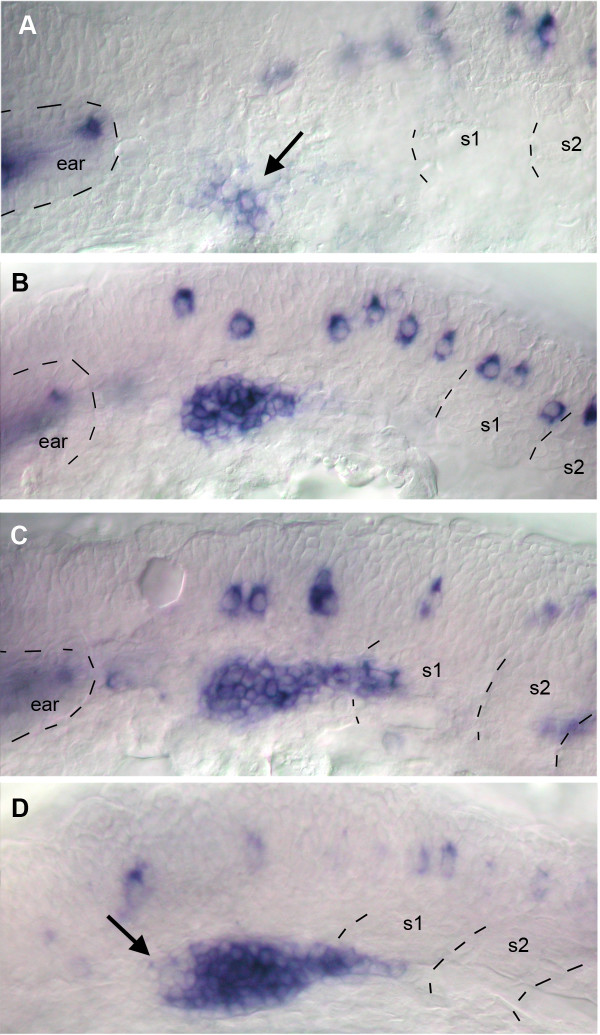
Early expression of *cxcr4b*. A: expression is first detected in a cluster of placodal cells (arrow) in 19 hpf embryos. B: expression is much enhanced in 20 hpf embryos. C: by 22 hpf the primordium has made contact with the SDF1 trail and elongates into somite 1. D: at about the same time *cxcr4 *expression is down-regulated in a small cluster of cells near the presumptive trailing edge of the primordium (arrow).

Expression of *cxcr4b *can already be detected at 19 hpf in a few cells of the placode (Fig. 5A, arrow). Expression quickly increases (Fig. 5B) and by the time the primordium begins to extend along the myoseptum (around 22hpf) all cells of the primordium express *cxcr4 *(Fig. 5C). Soon after, however, *cxcr4b *appears to be down-regulated in the trailing cells (Fig. 5D, arrow).

A comparison of the profiles of *cxcr7 *and *cxcr4b *around 22hpf suggests that *cxcr7 *is up-regulated and *cxcr4b *is down-regulated in the prospective trailing cells at the onset of migration. We cannot tell whether the up-regulation of *cxcr7 *and down-regulation of *cxcr4b *are exactly simultaneous, however, as there is some variability among embryos (e.g. the primordium is almost identical in shape and position in Figs. 5C and 5D, yet down-regulation of *cxcr4b *is evident in D but not in C) and double in situ hybridization is not as sensitive as single in situ in our hands.

**Figure 6:**
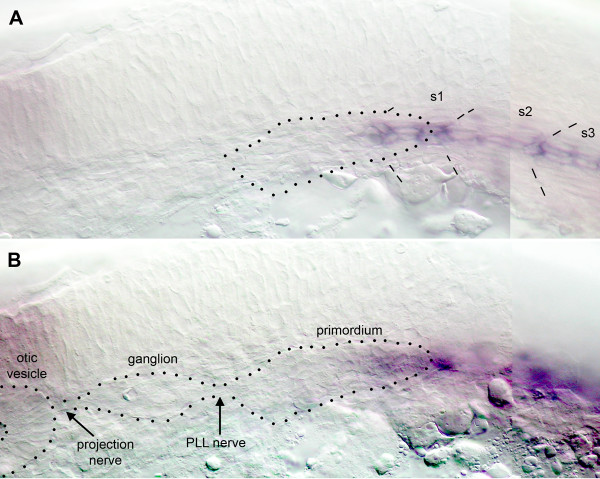
Early expression of *sdf1a *and primordium migration. A: At 22 hpf a thin stripe of cells along the horizontal myoseptum begins to express *sdf1a*. Dotted outline of the primordium derived from panel B. B: in the same embryo but in a slightly more superficial focal plane the primordium extends posteriorly and overlays the tip of the *sdf1a *stripe.

We also examined the expression of *sdf1a *at the onset of migration. At around 20 hpf *sdf1a *is expressed in a few cells at the anterior edge of the most anterior somites (not shown). Expression then extends to intervening cells such that a thin stripe of cells express the gene (Fig. 6A). At the same time *sdf1a *expression quickly extends to more posterior somites leading to a continuous stripe of *sdf1a *expression all along the horizontal myoseptum [11]. From the beginning of the process, the caudalmost somites express *sdf1a *in a more ubiquitous manner. The primordium begins to migrate and becomes separated from the ganglion at about 22 hpf (Fig. 6B). At this early stage its leading edge is already closely apposed to the cluster of *sdf1a*-expressing cells at the anterior edge of somite 1 (Fig. 6A, the outline of the primordium is taken from panel B).

### Inactivation of *cxcr7 *alters the pattern of neuromasts

**Figure 7:**
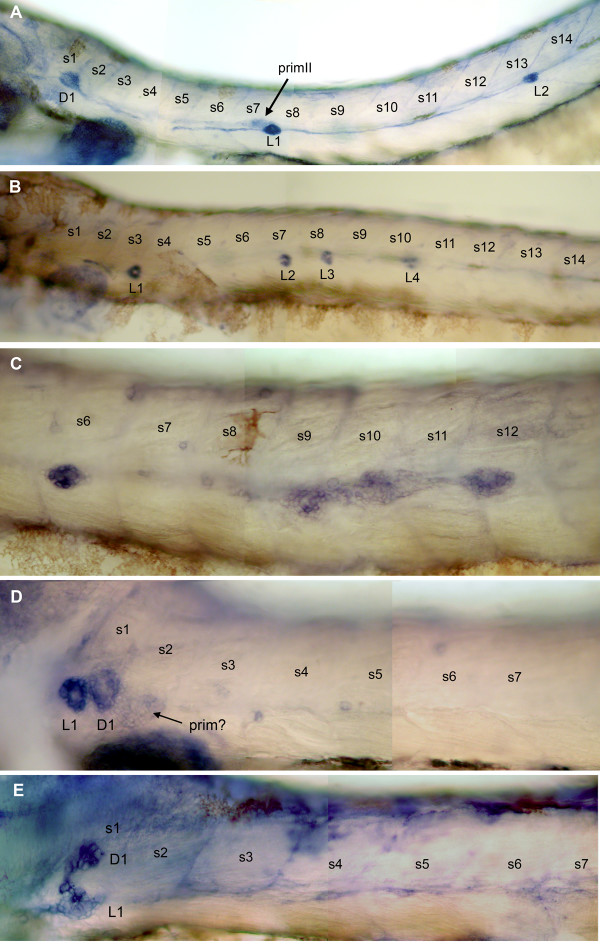
Morphant phenotypes. A: in wild-type 48 hpf embryos, alkaline phosphatase activity is present in the neuromasts, in the trail of interneuromastic cells and more weakly in primII (arrowed). The first neuromast of the dorsal line, D1, is already present at this stage. B: moderate phenotype in *cxcr7 *morphant embryos: there are fewer neuromasts (in this embryo, 4 instead of 7–8) and they are positioned closer together (see also Fig. 8). C: in about 10% of the cases the primoridum fragments in 2–3 clusters as is normally seen only for the terminal neuromasts at the tip of the tail. D: strong phenotype of a *cxcr7 *morphant embryo: no migration has taken place and there is a single neuromast, L1, at the level of the first somite. The first neuromast of the dorsal line, D1, has also formed. D1 can be unambiguously identified due to the anisotropy in alkaline phosphatase labeling which is orthogonal to that of the L neuromasts (panel B and [24]). A group of cells that may correspond to either primI or primII (arrowed) is stalled on somite 2.

In order to assess the function of *cxcr7 *in the migrating primordium, we inactivated the gene through injection of an antisense morpholino oligonucleotide (*cxcr7*-MO) at a concentration of 1.25 mM. At this concentration the survival rate is 95% and the embryos show no detectable delay in development or morphological abnormality. The embryos were labeled at 48 hpf for alkaline phosphatase. This enzyme is specifically expressed in the mature neuromasts and to a lesser extent in the undifferentiated cells of the PLL system, primordia and interneuromastic cells (Fig. 7A, [24]). Injection of a control morpholino oligonucleotide with 5 mismatches (see Material and Methods) resulted in 94% of the injected embryos (N = 87 sides for 52 injected embryos) showing a wild type pattern, 4% showing a developmental delay and 2% with a pattern reduced to two neuromasts.

**Figure 8:**
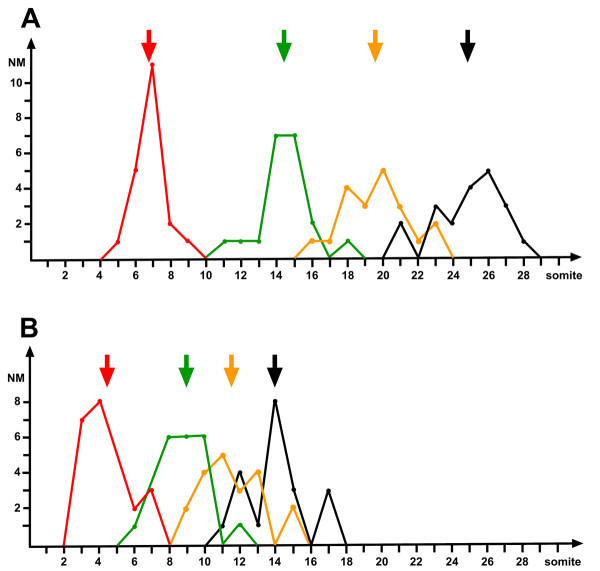
Distribution of the positions of the first four neuromasts, L1 to L4, in normal (A) and in *cxcr7 *morphant embryos (B). The large arrows indicate the average positions of L1–L4. The average positions of the neuromasts, based on 20 sides, were as follows: in the wild type (A): L1, 6.8 ± 0.9; L2, 14.4 ± 1.5; L3, 19.6 ± 1.9; L4: 24.9 ± 1.9. In the *cxcr7 *morphant (B), : L1, 4.3 ± 1.4; L2, 9.0 ± 1.3; L3, 11.5 ± 1.7; L4, 14.0 ± 1.7.

Among *cxcr7*-MO injected embryos, 87% were considered abnormal in that they lacked the terminal neuromasts, suggesting that migration was not completed. In 86% of the abnormal cases the number of neuromasts ranged from 0 to 4 instead of the normal 7–8 (Fig. 7B and Table 1). 31% had no neuromast at all beyond somite 2. One neuromast was usually present on somite 1 (Fig. 7D) adjacent to D1, the first neuromast formed by the secondary primordium [22]. In embryos with a reduced number of neuromasts, we observed that the distance between consecutive neuromasts is much reduced (Fig. 8) and it is not exceptional to find neuromasts on adjacent somites (Fig. 7B). Even though the distance between consecutive neuromasts is strongly reduced, the patterning remains rather normal, with a similar dispersion around average positions (Table 2). This suggests that the mechanism that determines the cyclic process of deposition is not altered in the morphant, but that a decreased rate of migration results in closer depositions.

**Table 1:** Numbers of neuromasts in morphant embryos

	no ter	0–2 NM	3–4 NM	5–6 NM	N
*cxcr7*-MO	63	48%	38%	14%	73
*cxcr4b*-MO1	11	100%	0%	0%	29
*cxcr7*-MO + *cxcr4b*-MO1	28	71%	18%	11%	38
*cxcr4b*-MO2	109	56%	28%	16%	123
*cxcr7*-MO + *cxcr4b*-MO2	74	53%	26%	22%	107

N: total number of embryos, no ter : number of embryos showing no terminal neuromasts and considered to be abnormal, 0–2, 3–4 and 5–6 NM: proportion of abnormal embryos presenting respectively 0–2, 3–4 and 5–6 neuromasts. Normal embryos present 7–8 neuromasts of which 5 are aligned along the myoseptum and 2–3 are present ventrally at the tip of the tail.

**Table 2:** Position of neuromasts in morphant embryos

	SD L1	SD L2	SD L3	SD L4	N
wild type	0.9	1.5	1.9	1.9	20
*cxcr7-*MO	1.4	1.3	1.7	1.7	20
*cxcr4b-*MO2	1.6	2.6	3.1	3.8	36
*cxcr7-*MO *+ cxcr4b*-MO2	1.9	1.6	2.2	2.4	23

The standard deviations around the average positions of L1, L2, L3 and L4 were compared for wild-type embryos and for morphant embryos that presented 4 neuromasts. N: number of sides that were used to calculate the standard deviations.

In wild type embryos, primI reaches the tip of the tail at about 40 hpf. There it fragments to form 2–3 closely apposed terminal neuromasts [7]. In morphant embryos at 48 hpf, the primordium is still visible in 90% of the cases, either at a very anterior position in the embryos where no or one neuromast has formed (Fig. 7D) or close to the last deposited neuromast in embryos where 2–5 neuromasts have formed. We occasionally observed 2 or 3 incompletely separated neuromasts (Fig. 7C), a pattern that is reminiscent of the fragmentation that takes place when primI has reached the tip of the tail in wild type embryos.

### Inactivation of *cxcr7 *affects primordium migration

The distribution of neuromasts along the antero-posterior axis is clearly affected in *cxcr7 *morphant embryos (Fig. 7). The reduced number and abnormal distribution of neuromasts suggest a defect in migration of the PLL primordium. Since the development of other structures (pectoral fins, eyes, ear and anterior lateral line) appears completely normal in *cxcr7 *morphants, the defect in migration does not result from a general impairment of development.

In order to confirm that migration is defective in the morphants we followed the course of the primordium under Nomarski optics. We examined 12 morphant embryos every 3 hours between 24 hpf and 36 hpf and determined the position of the leading edge of primI. We also determined the pattern of neuromasts at 48 hpf after alkaline phosphatase labelling. We observed that either the primordium does not migrate and extends no further than somite 2 at most (3 cases), or that it migrates at a reduced speed (9 cases). The speed varied between 0.2 somite and 0.7 somite per hour depending on the embryo, with an average of 0.4 ± 0.17 somite per hour. The speed in the wild type is 1.5 – 1.7 somite/hour. In the 9 embryos where migration was slowed down, the position reached by the primordium at 48 hpf was at most two somites beyond the position that the primordium occupied at 36 hpf, suggesting that migration stopped a few hours after 36 hpf, at about the time when migration stops in the wild type (40 hpf).

### Comparison of *cxcr4b*-MO and *cxcr7*-MO phenotypes

The gene *cxcr4b *is essential for proper migration of the primordium. Its pattern of expression fits well with this role, as it is highly expressed in the migrating cells of the primordium and less so in the trailing cells which are beginning to slow down. We have shown that the gene *cxcr7 *is also required for proper migration, yet its pattern of expression is opposite to that of *cxcr4b *as it is highly expressed in the cells that are being deposited, and not at all in the actively migrating cells.

In order to determine whether there is some interaction between *cxcr4b *and *cxcr7*, we first compared the phenotypes of *cxcr4b*-MO, of *cxcr7*-MO and of double *cxcr4b-*MO, *cxcr7*-MO embryos at 48 hpf. We used two different *cxcr4b *morpholinos, as discussed in Methods, one with a low survival rate, low penetrance and very high expressivity (*cxcr4b-*MO1, [11]) and one with a much higher survival rate and penetrance but a lower expressivity (*cxcr4b-*MO2, [12]). Since the phenotypes produced by the two morpholinos are somewhat different, we will discuss the results separately.

The phenotype of embryos injected with *cxcr4b-*MO1 is very similar to the strongest phenotype of *cxcr7-*MO, with one or two neuromasts around somite 1 (Fig. 7E and Table 1). We did not observe the intermediate phenotypes that are often present in *cxcr7 *morphants, with 1–4 neuromasts extending between somites 2 and 15 approximately. Interestingly, the severity of the *cxcr4b-*MO1 phenotype is largely relieved by the simultaneous inactivation of *cxcr7 *in double morphant embryos. In this case, up to 30% of the affected embryos show intermediate phenotypes that are typical of the *cxcr7 *morphants (Table 1). In many affected embryos the stalled primordium is still visible at 48 hpf after alkaline phosphatase labelling. We observed that the primordium reaches the posterior half of the body in 38% of the double morphant embryos (N = 32), very similar to the proportion in *cxcr7-*MO embryos (33%, N = 45). The primordium never extends beyond somite 5 in embryos that are injected with *cxcr4b-*MO alone. It appears, therefore, that the expression of *cxcr7 *aggravates the effect of *cxcr4b *deprivation. We conclude that CXCR7 may have an antagonistic role to that of CXCR4 in the primordium, consistent with their complementary patterns of expression.

The phenotype of embryos injected with *cxcr4b-*MO2 is milder than that of *cxcr4b-*MO1 morphants and resembles that of *cxcr7 *morphants (Table 1). The phenotype of the double *cxcr4b, cxcr7 *morphant is very similar to that of single *cxcr4b-*MO2 and *cxcr7-*MO injected embryos. Intriguingly, however, we had the impression that the pattern of neuromasts in the *cxcr4b-*MO2 morphant is more irregular that in either the *cxcr7 *or the double morphant. We quantified this impression by determining the standard deviation of the positions of L1 to L4 in all cases where only four neuromasts were present (Table 2). The results show that the pattern is substantially more irregular in *cxcr4b-*MO2 embryos than in either the *cxcr7 *or the double morphant, suggesting that the expression of *cxcr7 *in the presence of reduced levels of CXCR4 makes migration more erratic.

### Expression of *cxcr4b *in *cxcr7*-MO embryos

**Figure 9:**
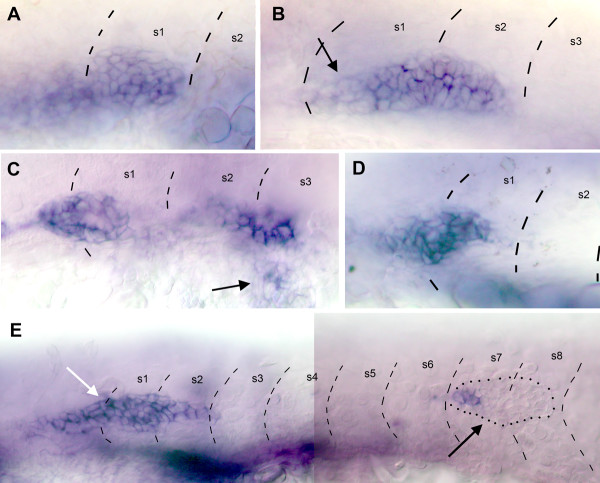
Expression of *cxcr4b *and *cxcr7 *in morphant embryos. A, B: expression of *cxcr4b *in 32hpf Mo-*cxcr7 *embryos. A: the shape of the stalled primordium is usually round and expression of *cxcr4 *appears ubiquitous. B: in a primordium that has migrated over a few somites, the expression of *cxcr4b *is reduced in the trailing cells (arrow), but the expression appears less asymmetric than in wild type embryos (compare with Fig. 3C). C, E: Expression of *cxcr7 *in 32hpf *cxcr4b-*MO1 embryos. C: in this embryo part of the primordium has reached somite 2–3 and has begun to extend over the yolk (arrow). All cells express *cxcr7*. E: an exceptional case where half of the primordium has remained around somite 1 and the other half has migrated up to somite 8 (in a normal embryo the primordium would have reached somite 15–20 at this age). In the stalled group all cells express *cxcr7 *while in the migrating group only the trailing cells express this gene. D: inactivation of SDF1 leads to the stalling of the primordium and to the expression of *cxcr7 *in all cells. Dashed lines indicate the positions of somite borders and dots in panel E show the outline of the primordium, as seen under Nomarski optics.

The lack of migration in *cxcr7-*MO embryos might be due, not to a requirement for the gene in the trailing cells of the primordium, but to an earlier expression of *cxcr7 *that would be necessary for the onset of *cxcr4b *expression. We did not detect such an early expression but decided nevertheless to see if the expression of *cxcr4b *is altered in *cxcr7-*MO embryos. As shown in Fig. 9A, *cxcr4b *is expressed in the primordium of 32 hpf *cxcr7 *morphants. This indicates that the absence of migration in *cxcr7 *is unlikely to result from a down-regulation of *cxcr4b*.

The non-migrating primordium usually assumes a round shape ([8], Fig. 9A) and does not show any clear heterogeneity or asymmetry in the expression of *cxcr4b*, suggesting that CXCR7 plays a role in establishing or maintaining the asymmetry of *cxcr4b *expression. In cases where the primordium shows abortive migration and reaches somites 2–5, the expression of *cxcr4b *is lower in the trailing cells than in other cells (Fig. 9B, arrow), suggesting that the asymmetry in *cxcr4b *expression does not entirely depend on the presence of CXCR7 in the trailing region. Even in this case, however, the asymmetry in *cxcr4b *is not as pronounced as in a normally migrating primordium (compare Fig. 9B and 3C). We conclude that the presence of CXCR7 in the trailing cells contributes to the down-regulation of *cxcr4b*. This conclusion must remain tentative because the expression of *cxcr4b *is dynamic: in normal conditions the expression is more homogeneous after deposition and more asymmetrical prior to deposition, complementary to the pattern of expression of *cxcr7 *(Fig. 3A, 3B).

### Expression of *cxcr7 *in *cxcr4b*-MO and in *sdf1a*-MO embryos

Given the complementarity in the patterns of expression of *cxcr7 *and *cxcr4b *we also examined the expression of *cxcr7 *in the non-migrating primordium of *cxcr4b *morphants (Fig. 9C, 9E). The outlines of non-migrating primordia are not as distinct under Nomarski optics as those of normal primordia but it appears clearly that most or all primordium cells express *cxcr7 *in morphant embryos (Fig. 9C; in this embryo part of the primordium has reached somite 2–3 and extends over the yolk, arrow). In another revealing case (Fig. 9E), one half of the primordium has remained paralysed around somite 1, while the other half has migrated (although at a reduced pace), suggesting that there was enough residual expression of *cxcr4b *in those cells to ensure some migration. All cells of the stalled group express *cxcr7*. Within the migrating group *cxcr7 *is expressed exclusively in the trailing cells. We conclude that the expression of *cxcr4b *is required to confine *cxcr7 *expression to the trailing region of the primordium.

If repression of *cxcr7 *in the leading region of the primordium depends on the activity of CXCR4, one would expect to observe ubiquitous expression of *cxcr7 *not only in the absence of CXCR4, but also when CXCR4 activation is prevented by the absence of its ligand. We examined the expression of *cxcr7 *in *sdf1a*-MO embryos [11]. We observed a ubiquitous expression of *cxcr7 *in stalled primordia (Fig. 9D) much as in the case of *cxcr4b *morphants. We also saw two cases of split primordium similar to the case shown Fig. 9E.

### Effect of *cxcr7 *inactivation on the formation of the secondary lateral line

We examined the effect of *cxcr7 *inactivation on the migration of primII at 2 and 6 days. At 2 days, primII is located between somites 4 and 7 in wild type embryos. In *cxcr7-*MO embryos of the same age, we observed that migration of primII is affected and that the severity of this effect is correlated with the severity of the effect on primI migration: no migration when primI is immobilized in the 0s-5s region, migration in 20% of the cases (2/10) where primI is stalled between 10s-15s, in 35% of the cases (5/14) when primI is found between 16s-25s and in 100% of the cases (N = 10) when primI migrates normally.

The primordium of the dorsal line originates together with primII and the two primordia split at about 36 hpf [22]. The dorsal primordium deposits the first neuromast of the dorsal line, D1, shortly thereafter. We observed that in *cxcr7 *morphants neuromast D1 is present in all cases, suggesting that the secondary primordium forms normally (Fig. 7D). The same result is observed in *cxcr4b *morphants (Fig. 7E).

We verified this result in 6 days-old larvae where primII has deposited 3–4 neuromasts and the dorsal line also comprises 2–3 neuromasts. Secondary PLL neuromasts can be distinguished from primary neuromasts by their polarization which is revealed by anisotropic alkaline phosphatase labelling. Among 42 severely affected *cxcr7-*MO embryos with no primary neuromast or one neuromast on somite 1, only 2 had formed a secondary neuromast, but all of them had developed a normal dorsal line (not shown), supporting the idea that the inactivation of *cxcr7 *affects specifically the migration of primII.

Since *cxcr7 *is not detectably expressed in primII, the easiest explanation for the lack of primII migration in morphant embryos is that primII relies on a trail left by primI (possibly the nerve, or the interneuromastic cells) such that if primI does not migrate neither can primII. The effect of *cxcr7 *inactivation on primII migration would therefore be indirect. We cannot, however, exclude the possibility that *cxcr7 *is transcribed in primII at such a low level that its expression would escape detection by in situ hybridization.

## Discussion

### Migration as a collective process

The zebrafish lateral line is emerging as an attractive system to study programmed cell migration. A number of studies have conclusively demonstrated that in this system migration depends on the interaction between the chemokine SDF1, which labels the path of migration, and its receptor CXCR4, which is present on the migrating cells [8,11,12]. SDF1/CXCR4 interactions also underly other long-range migration events such as the movement of germ cells both in fish [25,26] and in mouse [27], the migration of facial motoneurons in fish [28] and the movement of tumor cells in the formation of metastases [29].

Much has been learned about the implication of the SDF1/CXCR4 system in cell migration in the immune system, where cells seem to behave independently of each other. In the case of the PLL, however, cells move as a disciplined cohort and act in a coordinated manner. They keep their relative positions during migration and the cells that are deposited are always the trailing cells of the primordium [7,8]. In the case of the germ cells, cells remain in contact during their migration although they do not show the stable organization of the primordium cells [30]. In the case of cancer cells, collective or cohort migration has also been documented [31].

### *cxcr7 *and primordium migration

The gene *cxcr4b *is expressed in all cells of the primordium but its level of expression is lower in the trailing cells, consistent with the fact that those cells will soon slow down and stop migrating. Thus the pattern of expression of *cxcr4b *fits perfectly with an active role in cell migration. In this paper we describe the expression of the gene that encodes another chemokine receptor, CXCR7. The gene *cxcr7 *is expressed in the primordium in a pattern that is complementary to that of *cxcr4b*. Thus *cxcr7 *is maximally expressed in the cells that will be deposited next, and not at all in the actively migrating cells of the leading half of the primordium. It came as a surprise, therefore, to find that the inactivation of *cxcr7 *blocks migration much as the inactivation of *cxcr4b*. We heard from Darren Gilmour, at a recent meeting (15–18 March 2007, Minerve, France) that he has obtained very similar results about the expression and inactivation of *cxcr7*.

A cue to the function of *cxcr7 *comes from the analysis of the simultaneous inactivation of *cxcr7 *and of *cxcr4b*, as compared to the inactivation of *cxcr4b *alone. The phenotype of *cxcr4b *morphants, where *cxcr7 *is active, is substantially stronger than the phenotype of the double morphant, where *cxcr7 *is not active. We conclude that in conditions of reduced *cxcr4b *expression, the expression of *cxcr7 *has a negative effect on the residual migration of the primordium, consistent with its expression in the cells that are slowing down in wild type embryos.

A second cue to the function of *cxcr7 *comes from the observation that the inactivation of *cxcr4b *results in a deregulation of *cxcr7*, which becomes expressed in most or all cells of the primordium instead of being confined to its anteriormost (trailing) region. The down-regulation of *cxcr7 *by SDF1/CXCR4 in wild type embryos is consistent with the idea that the presence of CXCR7 in the leading cells would be detrimental for migration. Deregulation of *cxcr7 *in *cxcr4*b-MO embryos probably contributes to the aggravation of phenotype observed in *cxcr4b*-MO1 embryos vs the double *cxcr4b, cxcr7 *morphants.

How could a receptor that has a negative effect on migration be indispensable for migration? One obvious possibility is that CXCR7 is involved in defining the directionality of migration. Thus *cxcr7 *inactivation would not impair migration *per se*, but would make it impossible for the primordium cells to move coherently in one direction, thereby resulting in stalling of the direction-less primordium.

### *cxcr7 *and directionality

The PLL primordium migrates consistently from anterior to posterior even though the expression of *sdf1 *appears constant along the myoseptum. This suggest that the primordium has an intrinsic polarity, with a "plus" end at its leading edge and a "minus" end at the trailing edge. The idea that the primordium is intrinsically polarized is supported by experiments showing that a primordium confronted to an interruption in the SDF1 trail will sometimes make a U-turn and follow the trail of SDF1 in the opposite direction, towards the head [8]. Furthermore, it has been shown that a few wild-type cells at the leading edge are sufficient to rescue the migration of the entire primordium when *cxcr4*b is inactivated [8]. These results demonstrate that an intrinsic asymmetry in *cxcr4b *expression underlies the directionality of primordium migration. Our results suggest that this intrinsic asymmetry depends at least in part on the localized expression of *cxcr7 *in the trailing region and/or on its absence in the leading region.

**Figure 10:**
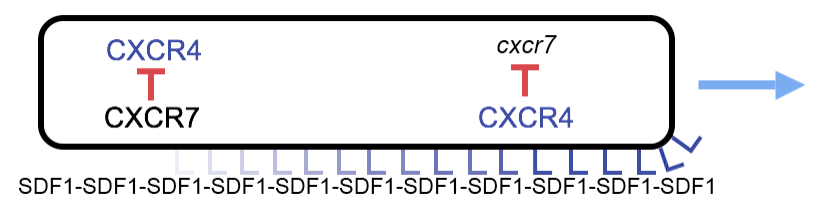
Cartoon illustrating the gradient of CXCR4 signalling that drives the primordium to migrate towards the right even if the concentration of SDF1 along the trail is constant. In the leading region (right part of the primordium) CXCR4 is present at high levels and is activated by binding to its ligand SDF1, resulting in active migration and repression of *cxcr7*. In the trailing region (left part) the high level of CXCR7 masks or sequesters the ligand, making it unavailable for binding and thereby preventing activation of CXCR4. Not mentioned in this cartoon are the possibility that SDF1 internalization will progressively reduce the concentration of ligand as the primordium moves along, and the possibility that the expression of *cxcr4b *depends at least in part on CXCR4 signalling, in which case *cxcr4b *would be down-regulated whenever CXCR7 is present at high levels.

What could be the role of *cxcr7 *in polarizing primordium migration? We suggest that the expression of *cxcr7 *defines the "minus" end of the primordium, such that even though the distribution of SDF1 is uniform along the pathway the primordium will move in the direction of its "plus" end. CXCR7 could define this "minus" end by maintaining or amplifying an early asymmetry in the activity of CXCR4 and/or expression of *cxcr4b *(see below for a possible origin of this asymmetry). Directional migration would then ultimately depend on the asymmetry in CXCR4 activity as cartooned in Fig. 10. In this model, directionality depends not on a gradient of SDF1 concentration but on a gradient in SDF1/CXCR4 signalling.

An antagonistic effect of CXCR7 on CXCR4 activation could be based on the high affinity of CXCR7 for SDF1, ten times higher than the affinity of CXCR4 for the same ligand [14]. The efficient binding of SDF1 to CXCR7 would lead to a masking or sequestering of SDF1 in the trailing region of the primordium, thereby making it unavailable to CXCR4. Furthermore data obtained in other systems suggest that the activation of CXCR4 may positively control the expression of the *cxcr4 *gene. The activation of CXCR4 by SDF1 promotes the formation of NFκB [32], which itself can induce the expression of *cxcr4 *[33]. We have evidence that this positive feedback loop is active in the PLL primordium (J. Torgersen, CDC, NC and AG, in preparation). The feedback loop would be interrupted when SDF1 is sequestered or masked, thereby leading indirectly to a reduction in *cxcr4b *expression. Thus an antagonistic effect of CXCR7 on CXCR4 activity may be achieved at two levels: first by sequestering its ligand SDF1, second by preventing the self-activation of *cxcr4b*. We cannot rule out, of course, that in addition to an inhibition of CXCR4 signalling, CXCR7 activation by SDF1 also has a more direct effect on migration directionality.

In morpholino conditions, reduced levels of *cxcr4b *expression may result in fluctuations in the cellular concentration of CXCR4, with some cells having a higher or lower concentration relative to their companions. The cells with a higher residual level of CXCR4 may then take the lead and the cells with a lower level may end up in the trailing region [8], thereby re-establishing some level of polarity and allowing some migration even if *cxcr7 *is not expressed. This would explain why, depending on the strength of *cxcr4b *morpholino inactivation, the expression of *cxcr7 *may either prevent migration altogether (*cxcr4b-*MO1), or simply make it more erratic (*cxcr4b*-MO2).

### Origin of primordium polarization

How could the anisotropy in the expression of *cxcr4b *and *cxcr7 *be initiated? We have observed that *cxcr4b *is expressed before the onset of migration, while *cxcr7 *is expressed later on. As the primordium splits from the ganglion and elongates, its most posterior cells come in contact with the stripe of SDF1 (which extends along the horizontal myoseptyum but not into the head). Activation of CXCR4 by SDF1 will induce migration of these cells along the SDF1 track, bringing the next cells in contact with SDF1. The migration of more and more cells along the myoseptum will lead to a progressive depletion of SDF1 through internalization of the ligand-receptor complex. Thus the last cells to come in will have a reduced level of CXCR4 activation, thereby allowing *cxcr7 *to become expressed. This would establish an early anisotropy of the primordium which would then be maintained due to the negative effect of *cxcr4b *on *cxcr7 *expression in the leading cells, and to the reciprocal negative effect of CXCR7 on CXCR4 function (through masking of SDF1) in the trailing cells.

Such a stable anisotropy would explain why, when a primordium turns back due to an interruption in the trail of SDF1, the cells do not simply go the other way around but the entire primordium doubles upon itself in a spectacular U-turn, such that its leading region will remain at the leading edge [8]. The fact that this turn is observed in only one tenth of the cases is consistent with the idea that the guiding trail has been at least partly depleted of SDF1 through binding and internalization of the ligand by both CXCR4 and CXCR7.

The migration of primordium cells as an organized cohort [8] may thus be in itself sufficient to generate its own directionality, since the concentration of SDF1 available to trailing cells will necessarily be lower than that available to leading cells (due to ligand/receptor internalization). CXCR7 would contribute to the control of primordium migration by reinforcing and stabilizing this intrinsic directionality, thereby allowing the fast and reproducible journey that is the basis for PLL development.

## Conclusion

We propose that the directional migration of the PLL primordium is determined by an intrinsic asymmetry due to the reciprocal distribution of two chemokine receptors that recognize the same ligand, chemokine SDF1: CXCR4 at the leading edge of the migrating primordium, and CXCR7 at its trailing edge. The interplay between the two receptors ensures that a constant distribution of SDF1 along the pathway is translated as a graded distribution of activated CXCR4 along the primordium, forcing primordium cells to move in the direction of higher SDF1/CXCR4 signalling, that is, in the direction of the leading cells and away from the trailing cells. The reciprocal expression of the two receptor genes is maintained through antagonistic interactions, and may originate automatically at the onset of migration due to the presence of the SDF1 stripe at one side of the newly born primordium.

## Methods

### Fish

Zebrafish (Danio rerio) were obtained from Singapour through a local company, Antinea, and maintained in standard conditions [35]. Embryos were obtained from pairs of adult fish by natural spawning and raised at 28.5°C in tank water. Ages are expressed as hours post fertilization (hpf).

### Identification of *cxcr7*

The gene *cxcr7 *was initially identified as an EST (cb900) and selected on the basis of its expression pattern [16]. This EST corresponds to gene si:dkey-96h14.2. Sequence alignment with mammalian genomes (*Homo sapiens, Mus Musculus and Rattus norvegicus*) revealed that this gene codes for the fish homolog of the chemokine receptor CXCR7.

### Morpholino knockdown experiments

Morpholinos oligonucleotides (Gene Tools, USA) were dissolved at 1.25 mM in 0.2 mM KCl and injected at the one-cell stage. This concentration gave the best combination of survival and phenotype. When 2 morpholinos were injected simultaneously, the solution contained each morpholino at a concentration of 1.25 mM. The antisense Morpholino sequences were designed to inhibit the translation of *cxcr7 *or *cxcr4b *mRNA. The MO-*cxcr7 *sequence is: 5'TCATTCACGTTCACACT**CAT**CTTGG-3'. The control morpholino had the following mismatches (underlined) : 5'TCATACACCTTGACACA**CAT**CTAGG-3'.

In the case of *cxcr4b*, two Morpholinos sequences were used: MO1-*cxcr4b *(5'- ATGATGCTATCGTAAAATTC**CAT**TT-3', [11]) and MO2-*cxcr4b *(5'-AAATGATGCTATCGTAAAATTC**CAT**-3', [12]). The bold CAT corresponds to the ATG translation start codon. As noted previously [11], MO1-*cxcr4b *morphants have a low penetrance (about 40% of injected embryos show an abnormal phenotype) but a high expressivity (the abnormal embryos show an extreme phenotype). Inactivation of *sdf1a *was done as described previously [11].

### In Situ Hybridization

20 to 35 hpf embryos were manually dechorionated, fixed in PBS-4%PFA for 2 hr at room temperature, rinsed in PBS and in 100% methanol and kept at -20°C. They were then processed for in situ hybridisation as described in [7,36].

### Neuromast labeling with alkaline phosphatase

48 hpf embryos were dechorionated, fixed in PBS-4%PFA for 2 hr at room temperature, rinsed in PBS and, if necessary, kept at 4° in PBS for up to a week. They were then processed for alkaline phosphatase labeling as described in [24].

## Competing interests

The author(s) declare that they have no competing interests.

## Authors' contributions

CDC designed and performed all the experiments. NC took care of fish handling including morpholino injections. AG contributed to the conclusions and drafted the manuscript. All authors read and approved the final manuscript.
